# Development of the Croatian model of organ donation and transplantation

**DOI:** 10.3325/cmj.2013.54.65

**Published:** 2013-02

**Authors:** Stela Živčić-Ćosić, Mirela Bušić, Željko Župan, Gordana Pelčić, Martina Anušić Juričić, Željka Jurčić, Mladen Ivanovski, Sanjin Rački

**Affiliations:** 1 Department of Nephrology and Dialysis, Department of Internal Medicine, University Hospital Rijeka, School of Medicine, University of Rijeka, Rijeka, Croatia; 2 Institute for Transplantation and Biomedicine, Ministry of Health, Zagreb, Croatia; 3 Department of Anesthesiology and Intensive Care, University Hospital Rijeka, School of Medicine, University of Rijeka, Rijeka, Croatia; 4 Department of Social Sciences and Medical Humanities, School of Medicine, University of Rijeka, Rijeka, Croatia; 5 Health Care Center of Primorsko-Goranska County, Rijeka, Croatia

## Abstract

During the past ten years, the efforts to improve and organize the national transplantation system in Croatia have resulted in a steadily growing donor rate, which reached its highest level in 2011, with 33.6 utilized donors per million population (p.m.p.). Nowadays, Croatia is one of the leading countries in the world according to deceased donation and transplantation rates. Between 2008 and 2011, the waiting list for kidney transplantation decreased by 37.2% (from 430 to 270 persons waiting for a transplant) and the median waiting time decreased from 46 to 24 months. The Croatian model has been internationally recognized as successful and there are plans for its implementation in other countries. We analyzed the key factors that contributed to the development of this successful model for organ donation and transplantation. These are primarily the appointment of hospital and national transplant coordinators, implementation of a new financial model with donor hospital reimbursement, public awareness campaign, international cooperation, adoption of new legislation, and implementation of a donor quality assurance program. The selection of key factors is based on the authors' opinions; we are open for further discussion and propose systematic research into the issue.

Transplantation is a widely accepted and successful life-saving treatment providing the best therapeutic benefit for hundreds of thousands of patients (1). Unfortunately, many people die while awaiting an organ transplant. A global shortage of organs available for transplantation raises many bioethical concerns, including the dilemma how to allocate limited resources to an unlimited number of needs and thus offer a fair and equal access to organ transplantation to all patients. Great efforts have been made to increase organ donation worldwide, but with only a moderate success in most of the countries. In contrast with this general trend, Croatia has recently experienced a boom in organ donation and transplantation. In 2011, Croatia had the highest rates of utilized cadaveric donors, kidney transplantations, and liver transplantations in the world (2-5) (Figure 1).

**Figure 1:**
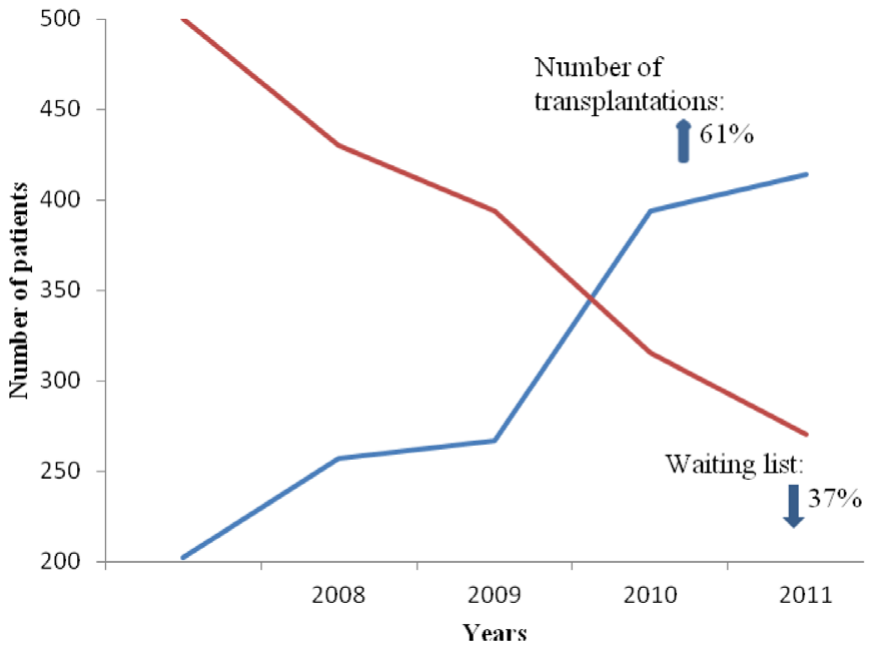
The number of kidney transplantations and the number of patients on the waiting list in Croatia between 2008 and 2011.

Remarkably, only one decade ago, Croatia was lagging far behind other European countries with a low donation rate (2.7 donors per million population [p.m.p.] in 2000). The continuous improvement of the organization of the Croatian transplant program resulted in a steadily growing donor rate, which reached the highest level in 2011, with 33.6 utilized donors p.m.p (2). We analyzed the factors that might have contributed mostly to this great success.

## Healthcare system

### Healthcare professionals

The increase in organ donation and transplantation rates in Croatia was primarily achieved by each hospital’s appointing an in-house transplant coordinator/key donation person (6). This has resulted in an improved detection and management of potential donors. Hospital transplant coordinators are well experienced and highly skilled intensivists, part time dedicated to the organ donation process. Most of them have been additionally trained in organ donation management at internationally recognized and licensed training courses for transplant coordinators (eg, Transplant Procurement Management Course).

Further factors were improvements and implementation of standardized protocols for optimal donor management, together with a proactive approach and early identification of all potential donors. Also, timely referral of a potential donor and aggressive donor management were implemented (7).

Professional and compassionate communication with the potential donor’s family has been shown to be crucial in lowering the refusal rate for organ donation (8). After confirmation of the patient’s death, family members are approached by the treating intensivist and the transplant coordinator in a professional and compassionate way, offering psychological support.

Improved family approach skills in Croatia have decreased the refusal rate for organ donation to less than 20% (data available from the Ministry of Health). In the Rijeka region, annual utilized donor rates increased between the periods 1986-1990 and 2010-2011 from 26.5 to 52.5 pm p., and non-utilized donor rate due to family refusal decreased from 46.5% to 7.1% (9) (unpublished data from the Ministry of Health).

### National organization of the transplant program

Croatia has a population of 4.29 million, 5 transplant centers, and more than 20 acute care hospitals participating in organ procurement through a well-organized and highly efficient transplant coordinator’s network. A crucial step in the Croatian transplant program was the appointment of hospital transplant coordinators in 1999 and the national transplant coordinator in 2000 (located at the Ministry of Health). In the following years, the national coordinator formed a coordination team at the Ministry to attend the 24-hour duty desk, supporting the hospital coordinators and procurement teams. The hospital coordinator network consists of 30 in-house coordinators, intensive care specialists working in university and general hospitals all over the country. Hospital coordinators are under the direct supervision of the hospital medical director, but maintain their independence within the hospital. The hospital transplant coordinator reports potential organ donors to the national coordination office, which facilitates and coordinates the donation process and international cooperation. This has eliminated many obstacles and made the process more efficient compared to the time when the local transplant team was in charge of procurement management and organ allocation.

**Figure 2:**
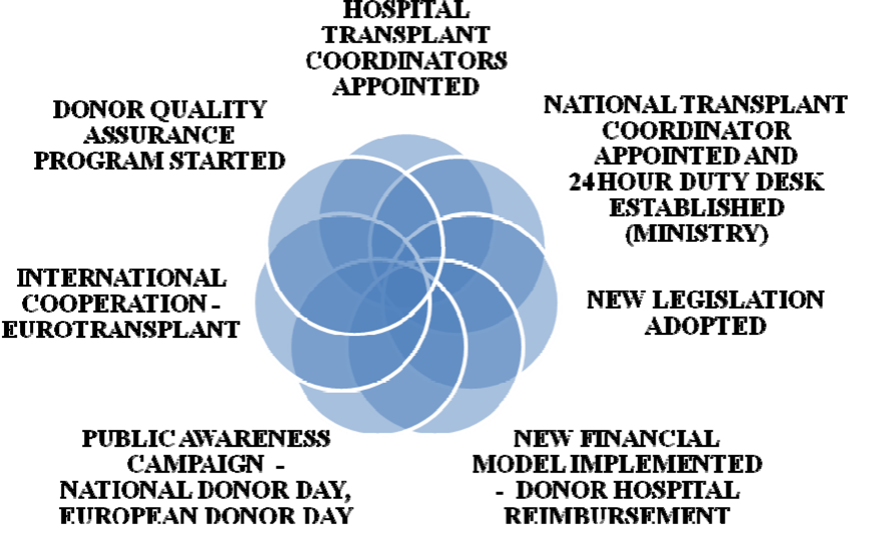
Identification of key factors for the increase of organ donation and transplantation rates in Croatia (2000-2010).

In 1999, the Croatian Parliament adopted the Resolution on Fostering Organ Transplantation, which emphasized the need for establishing a national organization for organ procurement and transplantation (10) and joining an international organization for organ exchange. The Ministry of Health implemented action plans, obtaining additional funds from the Croatian Government, and the Parliament was regularly informed about the implementation of the measures. The organ donation process in hospitals was supported by the Ministry of Health through close collaboration of the hospital coordinators’ network with the national transplant coordination team (Figure 2).

An increase in organ donation rate was followed by significant improvements in the waiting list management, pre-transplant evaluation, and screening of candidates for transplantation. Updated waiting lists of well-selected and carefully evaluated recipients enhanced the effectiveness of organ allocation and transplantation, strengthening the motivation and enthusiasm of transplant teams.

The reimbursement to donor hospitals implemented in 2006 partially compensated for the lack of financial resources, inadequate salaries, and shortage of health personnel in Croatia, which had been burdening the health system for a number of years, during which the transplant program had been kept alive mainly by the enthusiasm of the transplant team (5,11).

### International collaboration

A sustained increase in organ donation rate was one of the preconditions for the Croatian membership in the Eurotransplant (ET) (12). The Ministry of Health of the Republic of Croatia started negotiations with Eurotransplant International Foundation in 2006 and after one year of intensive preparations, Croatia became the seventh full member, and the only one outside the EU.

ET membership has provided a larger donor pool and thus better accessibility of organs, especially for patients in need for urgent transplantation. Every year, more than 7000 organs were allocated by ET using a point score system for an objective, fair, and transparent allocation. The ET membership contributed to the strengthening of public trust in the Croatian transplant program and fair organ allocation criteria.

Nowadays, the Croatian transplant program is successfully integrated into the ET Community and fulfills the requirements of the EU Directive 53/2010 on standards of quality and safety of human organs intended for transplantation (13).

Furthermore, a tremendous increase in donor rate prompted the European and world health community to recognize the “Croatian model” and there are plans for its implementation in other countries, especially in Southeastern Europe. Thus, in February 2011, within the Southeastern European Health Network, Croatia became a Regional Health Development Centre for organ donation and transplantation medicine in Southeastern Europe (14).

## Government and legislation

Since the adoption of the first legislation on the determination of brain death in 1982, cadaveric organ donation in Croatia has been almost exclusively based on donation after brain death. Donation after cardiac death had contributed only marginally and ended with the adoption of the new Croatian Transplant Act in 2004 (15). Furthermore, at that time transplantation from living donors was not recognized as a complementary method to transplantation from cadaveric donors, but rather as an exception. It has always been forbidden to advertise the need for transplantation, or the availability of organs or tissues/cells with an intention to offer or seek financial gain, as well as organ and tissue trafficking (15). Practically, almost all ethical principles that were defined in the Declaration of Istanbul and Convention on Human Rights and Biomedicine had already been implemented in the Croatian Transplant Act 2004, and transplantation clinical practice was harmonized with the state of the art of bioethical principles (16,17). During four decades of transplantation practice, not a single case of organ commercialism or malpractice was recorded.

Since 1988, the Croatian legal regulation for cadaveric donation has been based on a presumed consent (18). According to the law, body parts may be used for transplantation only if the deceased person did not make any written statement to be against organ donation (15). This “opt-out” system represents a practical and efficient legal framework for a successful deceased organ donation program (19). A non-donor registry has been implemented and maintained by the Ministry of Health. Currently, it includes less than 5‰ of Croatian citizens. If the deceased person is not registered in the non-donor registry, the family is informed about the possibility of organ donation and it is verified that the deceased person did not object to donation. The family’s decision is always respected. This kind of approach prevents negative publicity, which might lead to a reduction in the donor rate.

## The public

The key factors for a successful transplant program are a positive public attitude toward organ donation, solidarity, and willingness to help other people. Croatia has a long tradition of renal replacement therapy, ever since the beginning of dialysis treatment in 1962 and the first kidney transplantation in 1971 in Rijeka hospital, the leading kidney transplant center in the former Yugoslavia (20,21).

During the last decade, public awareness on organ donation has been improved through continuous education, donor card promotion, and national public campaigns organized by the Ministry of Health and non-governmental organizations (Croatian Donor Network, Croatian Transplant Association, Croatian Society of Nephrology, Dialysis and Transplantation, etc) (22-25). It has also been improved by better information availability, especially when information was provided by the professionals experienced in transplantation medicine (26) and by recipients who presented their life stories in the mass media (27,28).

The family’s opinion has a strong influence on the individual’s decision for organ donation (29) and the family’s opinion often depends on cultural and religious values and attitudes. Therefore, it is noteworthy that all religious communities in Croatia generally support organ donation and regard it as an altruistic and generous act. This positive attitude was confirmed by their readiness to promote organ donation in public through discussions, surveys, publishing papers, etc (30).

This continuous promotion influenced public opinion and attitude, as well as the increased respect of bioethical principles in the process of organ donation, allocation, and transplantation.

## Bioethical considerations

Organ donation is the ultimate gift that can be made by the family in mourning. Communication with the families of the deceased potential donors ensures that there are no doubts or open questions regarding confirmation of brain death or other issues related to organ donation. If all preconditions are fulfilled and the deceased person did not object to donation, the procedure is performed by respecting the highest standards, including the protection of the body and dignity of the deceased person. However, if the family is against organ donation, this will be always respected in accordance with bioethical principles.

The fundamental values and bioethical principles were endorsed by the Resolution on Fostering Organ Transplantation, passed by the Croatian Parliament in 1999 (10). Moreover, the higher standards than required by the Croatian law were defined in the Code of Ethics of the Croatian Medical Chamber (31). The practice was also in accordance with the high bioethical standards defined in the Additional Protocol to the Convention on Human Rights and Biomedicine, on Transplantation of Organs, and Tissues of Human Origin, which was adopted by the Croatian Parliament in 2003 (32).

In countries with a successful cadaveric donation program and short waiting time for transplantation, like Croatia, there is less need for living donations. The organ procurement procedure is harmful for the living donor and complications may arise even many years later. In addition, the outcomes after renal transplantation from the living and cadaveric donors have become very similar (33). This does not justify the risk of kidney transplantation from living donors in case of incompatible blood groups or positive cross-match between the donor and recipient. Our transplant program is based on the cadaveric donation, with a very low percentage of living donors, predominantly recipients’ relatives (2.0 donor p.m.p. in 2011) (2). In our opinion, there is not much room for improvement of our living donor program, mainly because of social and economic constraints (smaller potential pool of living-related donors due to smaller families, older population on dialysis, etc.). Altruistic living donation from unrelated individuals is allowed but not encouraged due to the bioethical considerations. In practice, medical and bioethical doubts often arise about the possibility to accept organs from extended criteria donors. The potential organ recipient must always be adequately informed and has to accept the transplantation procedure.

According to the law and bioethical principles, the basis of the Croatian transplant program will remain cadaveric donation as long as the number of donors is sufficient. Organ transplantation from living donors will be performed in individual cases, ensuring the respect of strict professional standards and bioethical criteria, after the approval of the institutional ethical committee.

## Conclusion

The key factors that have contributed to the development of a “successful model for organ donation and transplantation” in Croatia in the past decade are the appointment of hospital and national transplant coordinators, the establishment of a 24-hour duty desk at the Ministry of Health, and the implementation of a new financial model with donor hospital reimbursement. Public awareness campaign, intense international cooperation, accession to Eurotransplant, adoption of new legislation, and implementation of a donor quality assurance program also have greatly contributed to the success of our program.

The Croatian transplant program has resulted in a steep increase in organ donation and transplantation rates. These extraordinary results are primarily a consequence of a successful organizational model for organ donation and transplantation, the diligent work of devoted and enthusiastic health care professionals, respect for bioethical principles, and public solidarity and awareness of the benefits of organ transplantation. Also, this would not have been possible without altruism and unselfish help of families who, in moments of their personal tragedy, agreed to donate organs of their family members.
